# Caregiver Burden, Emotional Distress, and Coping Strategies in Romanian Parents of Children with Autism Spectrum Disorder: An Exploratory Cross-Sectional Comparative Study

**DOI:** 10.3390/diseases14060205

**Published:** 2026-06-08

**Authors:** Otilia-Rodica Butiu, Ema Burlacu, Rebeca-Isabela Molnar, Adriana Mihai, Teodora Popescu

**Affiliations:** 1 Psychiatry Department, George Emil Palade University of Medicine, Science, and Technology of Targu Mures, 540142 Targu Mures, Romania; otilia.butiu@umfst.ro (O.-R.B.);; 2 Neuropsychiatric Clinic, Mures County Hospital, 540005 Targu Mures, Romania; 3 Institute of Psychotherapy and Personal Development, 540038 Targu Mures, Romania; teodora.poprescu@umfst.ro; 4 Doctoral School of Medicine and Pharmacy, George Emil Palade University of Medicine, Science, and Technology of Targu Mures, 540142 Targu Mures, Romania; ema.burlacu@umfst.ro; 5 Psychiatric Clinic 1, Mures County Hospital, 540081 Targu Mures, Romania

**Keywords:** autism spectrum disorder, coping mechanisms, parents, comparative study

## Abstract

Background/Objectives: Parents of children with autism spectrum disorder (ASD) often face sustained emotional, practical, and social demands. However, evidence from Romania remains limited, particularly regarding the combined assessment of caregiver burden, emotional distress, and coping strategies of parents. This exploratory study compared these outcomes between parents of children/adolescents with ASD and parents of typically developing children and examined whether coping patterns varied according to selected sociodemographic characteristics. Methods: We conducted a cross-sectional comparative study in Târgu-Mureș, Romania, between 2024 and 2025. The sample included 92 parents: 46 parents of children/adolescents with clinician-confirmed ASD and 46 parents of typically developing children. Participants completed a demographic questionnaire, the Caregiver Burden Inventory (CBI), the Depression Anxiety Stress Scales-21 (DASS-21), and the Strategic Approach to Coping Scale (SACS). DASS-21 data were available for 44 ASD caregivers and 46 controls. Between-group comparisons were performed using *t*-tests, Mann–Whitney U tests, chi-square tests, or Fisher’s exact tests, as appropriate. Results: The groups were comparable in sex, age, residence, number of children, and household size, but differed significantly in marital status and educational level. Clinically relevant caregiver burden (CBI ≥ 36) was more frequent among parents of children with ASD than among controls (30% vs. 17%), although this difference was not statistically significant. Parents of children with ASD showed trend-level higher depressive and anxiety symptoms, with small effect sizes, whereas stress scores were similar between groups. Coping patterns varied according to sociodemographic characteristics. Marital status was associated with aggressive coping, urban residence was associated with indirect and aggressive coping, and number of children was associated with seeking social support. Conclusions: Parents of children with ASD showed a higher proportion of clinically relevant caregiver burden and trend-level elevations in depressive and anxiety symptoms, while stress scores were comparable between groups. Exploratory adjusted analyses suggested that ASD caregiver status remained associated with caregiver burden and depressive symptoms after controlling for educational level and marital status. Coping strategies appeared heterogeneous and context-dependent. Given the exploratory design, modest sample size, and multiple comparisons, these findings should be interpreted as preliminary and hypothesis-generating.

## 1. Introduction

Autism spectrum disorder (ASD) is a neurodevelopmental condition characterized by persistent difficulties in social communication and interaction, together with restricted, repetitive patterns of behavior, interests, or activities. Because ASD usually begins early in development and often requires long-term support, its impact extends beyond the diagnosed child and affects the daily functioning, emotional wellbeing, and social life of the family. Parents are frequently required to coordinate clinical appointments, educational support, behavioral interventions, and home-based strategies, while also managing uncertainty regarding the child’s developmental trajectory [1,2,3].

The increasing recognition and diagnosis of ASD have drawn attention to the psychological burden experienced by caregivers. International estimates suggest that ASD affects a substantial number of children worldwide, although prevalence varies by region, diagnostic practices, service availability, and surveillance methods [4,5,6]. These epidemiological data are clinically relevant because each diagnosis is accompanied by ongoing family adaptation. For many parents, caregiving involves not only practical demands, but also emotional strain, financial pressure, reduced personal time, and the need to advocate for the child across healthcare, educational, and social systems [1,2,3,7,8].

Previous studies have shown that parents of children with ASD often report elevated levels of caregiver burden, stress, depression, and anxiety compared with parents of typically developing children or parents facing other caregiving contexts [9,10,11,12,13]. These difficulties may be influenced by child-related factors, such as communication problems, behavioral dysregulation, sleep disturbances, intellectual disability, or comorbid psychiatric and neurological conditions. They may also be shaped by family and environmental factors, including social support, marital functioning, educational level, economic resources, residence, stigma, and access to specialized services [2,3,8,14,15,16]. Therefore, caregiver distress should not be understood as a simple or uniform reaction to the child’s diagnosis, but rather as the result of multiple interacting pressures.

Coping strategies are central to this process of adaptation. Coping refers to the cognitive, emotional, and behavioral efforts used to manage demands perceived as stressful or exceeding available resources. In families of children with ASD, coping may include problem-focused strategies, such as information seeking, planning, and active service navigation; emotion-focused strategies, such as acceptance, reframing, or religious coping; and avoidance or disengagement strategies, which may emerge when demands feel uncontrollable [17,18,19]. Although some strategies are generally considered more adaptive than others, their meaning depends on context. For example, seeking support may be protective when support systems are available, while avoidance may temporarily reduce emotional overload in situations where parents have limited control [15,16,17,18,19].

The social context in which families live is particularly important. Parents who have access to reliable information, specialized services, extended family support, and community understanding may be better positioned to use active and constructive coping strategies. In contrast, parents facing stigma, limited services, financial constraints, or social isolation may be more vulnerable to emotional distress and less adaptive coping patterns [2,3,8,15,16]. Sociodemographic variables such as marital status, educational level, urban or rural residence, and number of children may therefore influence both distress and coping. Cross-cultural and qualitative studies suggest that parental experiences are shaped by cultural expectations, family networks, stigma, spirituality, and community resources, which may differ substantially between countries and healthcare systems [2,3,20,21].

Another gap in the literature concerns the simultaneous assessment of caregiver burden, emotional symptoms, and coping strategies within the same comparative design. Many studies focus on general parental stress, while fewer examine depression, anxiety, caregiver burden, and specific coping profiles together [9,10,14,18,19]. Moreover, when comparison groups are included, sociodemographic differences between groups may complicate interpretation. Understanding these differences is important because caregiver outcomes may reflect not only the presence of a child with ASD, but also educational, marital, residential, and family-structure factors [16,22].

At present, ASD has no curative treatment; management relies on early, individualized, multimodal intervention and on sustained collaboration between families and specialized professionals [5]. This makes parents essential partners in care, but also increases the importance of monitoring their psychological wellbeing and coping resources. Identifying caregiver burden, emotional symptoms, and less adaptive coping patterns may help clinicians provide more timely and targeted support.

The present study therefore aimed to compare caregiver burden, emotional distress, and coping strategies in parents of children/adolescents with ASD and parents of typically developing children in Romania. A secondary aim was to explore associations between coping strategies and selected sociodemographic characteristics, including age group, marital status, education, residence, and number of children. Given the modest sample size and exploratory nature of the analyses, the study was intended to generate clinically relevant hypotheses rather than provide definitive causal conclusions.

## 2. Materials and Methods

### 2.1. Study Design and Setting

An exploratory cross-sectional comparative study was conducted in Târgu Mureș, Romania, between 2024 and 2025. The study was reported in accordance with the STROBE statement for observational studies.

### 2.2. Participants and Recruitment

Participants were recruited between 2024 and 2025 from the Pediatric Neurology and Psychiatry Clinic in Târgu Mureș, Romania. A non-probabilistic convenience sampling strategy was used. Parents/caregivers of children and adolescents with clinician-confirmed ASD were invited to participate during clinic visits. The ASD group included parents of children/adolescents aged 3–18 years with a diagnosis of ASD established by a specialist according to ICD-10 criteria (F84.0/F84.1/F84.5). The control group consisted of parents/caregivers of typically developing children recruited from the same clinical setting during the same period. Controls were eligible if their child had no known diagnosis of ASD or other neurodevelopmental disorder, based on caregiver report and available clinical information at enrollment. No individual matching procedure was performed; however, the two groups were compared descriptively and statistically for key sociodemographic characteristics. The final sample included 92 parents/caregivers: 46 in the ASD group and 46 in the control group.

Because recruitment was based on availability and willingness to participate, the sample should be interpreted as a clinical convenience sample rather than a representative sample of Romanian parents of children with ASD.

### 2.3. Exclusion Criteria

Exclusion criteria for both groups were: refusal or withdrawal of informed consent; insufficient questionnaire data to compute the relevant scale scores; inability to understand the study procedures or complete the questionnaires; and duplicate participation from the same parent/caregiver. For the control group, parents were additionally excluded if their child had a known diagnosis of ASD, intellectual disability, attention-deficit/hyperactivity disorder, developmental delay, or another neurodevelopmental disorder. Missing questionnaire data were handled using available-case analysis, and the effective sample size was reported for each analysis.

### 2.4. Measures

Participants completed a brief demographic questionnaire and three standardized self-report instruments:

#### 2.4.1. Caregiver Burden Inventory (CBI)

The CBI assesses multidimensional caregiver burden across domains including time-dependence, developmental burden, physical burden, social burden, and emotional burden. Total and subscale scores were computed according to the original scoring instructions.

#### 2.4.2. Depression, Anxiety and Stress Scales—21 Items (DASS-21)

The DASS-21 is a 21-item self-report instrument designed to assess depressive symptoms, anxiety, and stress over the previous week. The instrument includes three subscales corresponding to depression, anxiety, and stress. DASS-21 results were available as categorized symptom severity scores and automatically generated subscale scores provided by the psychological assessment platform. Analyses were therefore performed using these reported DASS-derived scores rather than individual item-level responses, which were not available for recalculation of standard DASS-21 total scores.

Psychological assessments and score generation were performed by a licensed psychologist using the Cognitrom Assessment System (CAS4 platform, Cognitrom, Cluj-Napoca, Romania), which provides standardized digital administration and automated scoring of validated psychometric instruments.

#### 2.4.3. Strategic Approach to Coping Scale (SACS)

The SACS evaluates coping strategies across multiple domains (e.g., assertive action, social support seeking, avoidance, indirect action, aggressive action). Subscale scores were computed according to the scale manual.

### 2.5. Data Collection Procedure

After eligibility confirmation, parents received written study information and completed the questionnaires in clinic. A member of the research team was available to clarify procedural questions, without influencing responses. Data were collected and stored anonymously using unique participant codes.

### 2.6. Ethics Approval

Written informed consent was obtained from all participants prior to data collection. The study was conducted in accordance with the Declaration of Helsinki and approved by the Ethics Committee of the Institute of Psychotherapy and Personal Development (decision no. 19/28 October 2023).

### 2.7. Statistical Analysis

Statistical analyses were performed using GraphPad InStat (GraphPad Software, San Diego, CA, USA version 11.0.2), and PSPP statistical software version 1.6.2. Continuous variables were summarized as mean ± standard deviation (SD) when approximately normally distributed; otherwise, median and interquartile range (IQR) were used. Normality was assessed using the Shapiro–Wilk test.

Between-group comparisons for continuous demographic and psychometric variables, including age, CBI scores, DASS-21 scores, and SACS subscale scores, were conducted using Student’s *t*-test for normally distributed variables and the Mann–Whitney U test for non-normally distributed variables. Categorical variables were compared using the chi-square test; Fisher’s exact test was applied when expected cell counts were <5. Categorical variables included sex, marital status, educational level, residence, and categorized burden levels. Associations between categorical variables were expressed as relative risks (RR) with 95% confidence intervals (CI), where applicable.

Exploratory multivariable linear regression analyses were additionally performed to adjust for potential confounding effects of educational level and marital status. Caregiver burden and DASS-21 subscale scores were included as dependent variables, while group status (ASD vs. control), educational level, and marital status were entered as independent variables. Regression assumptions were explored through inspection of residual distributions and multicollinearity diagnostics.

Missing data were limited and primarily reflected incomplete questionnaire responses. Analyses were conducted on an available-case basis, such that each scale analysis included participants with sufficient data to compute the corresponding score; effective sample sizes are reported where applicable.

No a priori sample size calculation was performed due to the exploratory nature of the study.

All tests were two-tailed, and statistical significance was set at α ≤ 0.05.

## 3. Results

### 3.1. Sociodemographic Characteristics

A total of 92 parents were included (ASD group: *n* = 46; control group: *n* = 46). Sex distribution was identical across groups (41 mothers [89%] and 5 fathers [11%] in each), with no between-group difference (Fisher’s exact test, *p* = 1.00; RR = 1.00, 95% CI: 0.52–1.93). Most parents were aged ≥35 years (ASD: 76% vs. controls: 63%), without a statistically significant difference (χ^2^ test, *p* = 0.257; RR = 1.21, 95% CI: 0.86–1.69)as seen in Table 1.

Marital status differed significantly between groups: 70% of parents in the ASD group were married compared with 91% in the control group (Fisher’s exact test, *p* = 0.0164; RR = 0.77, 95% CI: 0.62–0.96), indicating a higher proportion of single/divorced parents among caregivers of children with ASD. Residence showed a non-significant trend, with fewer urban participants in the ASD group (54%) than in controls (72%) (*p* = 0.130; RR = 0.75, 95% CI: 0.52–1.07). Educational attainment differed markedly: higher education was reported by 26% of ASD caregivers versus 76% of controls (χ^2^ test, *p* < 0.0001; RR = 0.34, 95% CI: 0.21–0.56).

No significant differences were observed in family composition. The distribution of number of children per family was comparable between groups (*p* = 0.932; RR = 1.03, 95% CI: 0.74–1.44), with two-child families predominating. Family size was also similar (*p* = 0.916; RR = 1.02, 95% CI: 0.73–1.43), most commonly four members (ASD: 46% vs. controls: 50%).

### 3.2. Caregiver Burden

Caregiver burden was assessed using the Caregiver Burden Inventory (CBI), a 24-item instrument measuring five domains: time-dependence, developmental, physical, social, and emotional burden. Items are rated on a 5-point Likert scale (0–4), and a total score ≥ 36 is used to indicate clinically relevant burden. In the present sample, 14 parents (30%) in the ASD group had CBI scores ≥ 36 compared with 8 (17%) in the control group, indicating numerically higher burden levels among ASD caregivers, although the difference did not reach statistical significance (Fisher’s exact test, *p* = 0.197; RR = 1.76, 95% CI: 0.82–3.77).

For clinical interpretation, in addition to the total CBI score, elevated responses at the item level (e.g., ratings ≥ 3) may help identify specific burden domains to target in individualized support, even when the overall score remains below the ≥36 threshold.

### 3.3. Emotional Symptoms (DASS-21)

Emotional symptoms were assessed using the Depression, Anxiety and Stress Scales–21 items (DASS-21), which evaluates depressive symptoms, anxiety, and stress over the previous week. The instrument includes three subscales corresponding to depression, anxiety, and stress. DASS-derived results were available as categorized symptom severity scores generated through the psychological assessment platform rather than recalculated standard DASS-21 total scores based on individual-item responses.

Parents of children with ASD showed higher depression and anxiety scores compared with controls, although between-group differences did not reach statistical significance. Depression scores were higher in ASD caregivers (1.80 ± 1.39) relative to controls (1.24 ± 0.79) (Mann–Whitney U = 795.5, *p* = 0.073; r = 0.18). Similarly, anxiety scores were higher in the ASD group (1.75 ± 1.14 vs. 1.26 ± 0.88; U = 778.5, *p* = 0.054; r = 0.20). In contrast, stress scores were comparable between groups (1.57 ± 1.17 vs. 1.48 ± 0.94; U = 990.5, *p* = 0.861; r = 0.02). Detailed results are presented in Table 2.

**Figure 1:**
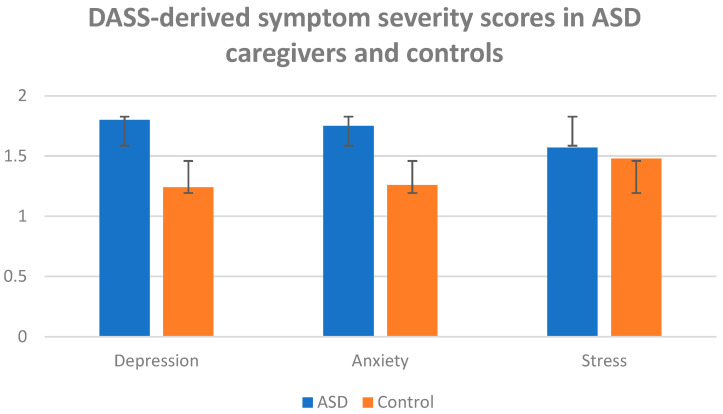
Mean DASS-derived depression, anxiety, and stress severity scores in parents of children with ASD and controls. Error bars represent standard deviations.

Mean DASS-derived depression, anxiety, and stress severity scores are presented in Figure 1.

Coping strategies were evaluated using the Strategic Approach to Coping Scale (SACS), developed within the Conservation of Resources framework and designed to capture behaviorally anchored coping responses in a social context. The instrument includes 52 items organized into nine subscales (e.g., Assertive Action, Avoidance, Social Joining, Seeking Social Support, Cautious Action, Instinctive Action, Indirect Action, Antisocial Action, Aggressive Action). The Romanian version has been previously adapted and psychometrically examined.

#### 3.3.1. Marital Status and Coping Patterns

A significant association was observed between marital status and aggressive coping. Married parents more frequently scored ≥40 on the Aggression subscale compared with unmarried parents (Fisher’s exact test, *p* = 0.044). Specifically, 29/32 (≈91%) married parents scored ≥40 on the Aggression subscale, versus 9/14 (≈64%) among unmarried parents. Married parents were 3.81 times more likely to score ≥40 on Aggression (RR = 3.81; 95% CI: 1.05–13.79; Katz approximation).

**Figure 2:**
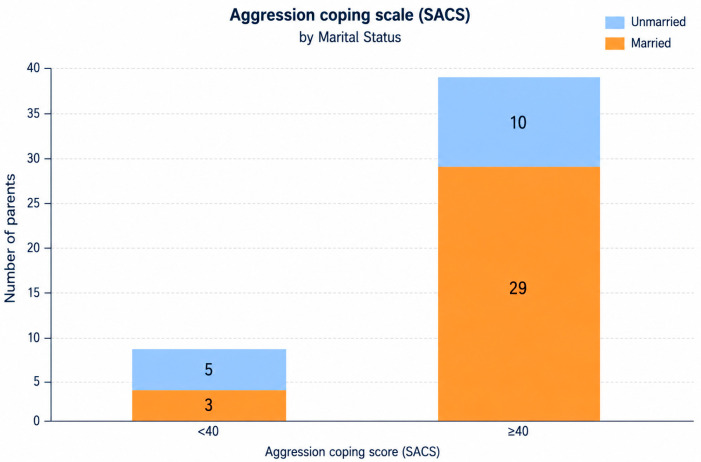
Distribution of aggressive coping according to marital status.

The distribution of aggressive coping according to marital status is shown in Figure 2.

A higher proportion of married parents scored ≥40 on the Instinctive coping subscale compared with unmarried parents (30/31 [≈97%] vs. 11/14 [≈79%]); however, the association did not reach statistical significance (*p* = 0.0825).

#### 3.3.2. Area of Residence and Coping Patterns

Area of residence was significantly associated with indirect coping. Parents living in urban areas more frequently scored ≥40 on the Indirect coping subscale compared with those in rural areas (22/25 [≈88%] vs. 11/21 [≈52%]) (*p* = 0.0102). Urban residence was associated with an approximately fourfold higher likelihood of indirect coping (RR = 3.97; 95% CI: 1.25–12.57).

**Figure 3:**
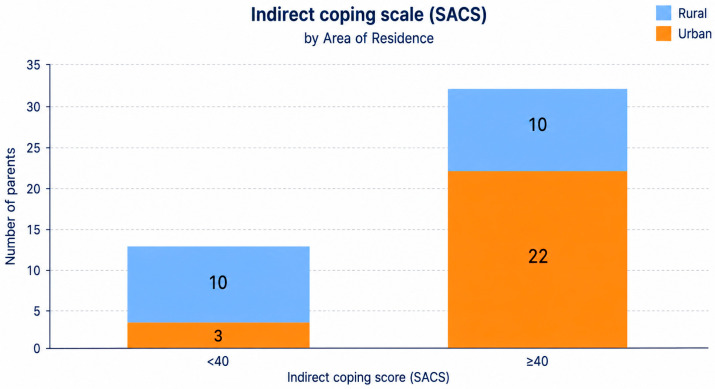
Distribution of indirect coping according to area of residence.

The distribution of indirect coping according to area of residence is shown in Figure 3.

A significant association was also found between residence and aggressive coping. Parents from urban areas were more likely to score ≥40 on Aggressive coping than rural parents (Fisher’s exact test, *p* = 0.0161), corresponding to a large relative risk estimate (RR = 8.33; 95% CI: 1.11–62.43; Katz approximation).

A non-significant association was also observed between residence and stress (*p* = 0.0839), with urban parents tending to report higher stress levels.

#### 3.3.3. Family Context and Social Coping

The number of children was significantly associated with social coping (seeking social support) (*p* = 0.0444). All parents with one child scored ≥40 on the Seeking Social Support subscale, whereas variability was present among parents with two or more children (9 scored <40 and 24 scored ≥40). Because all parents in the single-child group scored ≥40, a relative risk estimate could not be reliably computed.

#### 3.3.4. Between-Group Differences on Social Coping and Social Relations

On the SACS Seeking Social Support subscale, a statistically significant between-group difference was identified (Mann–Whitney U = 733.5, *p* = 0.0114). Parents of children with ASD reported higher scores (49.26 ± 10.47) compared with controls (44.02 ± 8.94), suggesting greater use of support-seeking coping strategies as seen in Table 3.

Social Relations scores did not differ significantly between groups (Mann–Whitney U = 1048.0, *p* = 0.9408).

Across analyses, coping strategies varied as a function of parental characteristics. Significant associations were identified for marital status (aggressive coping), area of residence (indirect and aggressive coping), and number of children (seeking social support), while non-significant associations were observed for marital status (instinctive coping) and residence (stress). These findings suggest variability in coping profiles according to parental sociodemographic characteristics.

Because educational level and marital status differed significantly between groups, exploratory multivariable regression analyses were performed to assess whether ASD caregiver status remained independently associated with caregiver burden and psychological symptom measures after adjustment for these potential confounders.

Exploratory multivariable regression analyses demonstrated independent associations between ASD caregiver status and both caregiver burden and depressive symptom scores after adjustment for educational level and marital status, as seen in Table 4. Anxiety symptom scores also remained significantly associated with ASD status at the predictor level, although the overall regression model did not reach conventional statistical significance. In contrast, no independent association between ASD caregiver status and stress scores was identified after adjustment.

## 4. Discussion

The present exploratory study examined caregiver burden, emotional distress, and coping strategies among Romanian parents of children/adolescents with ASD compared with parents of typically developing children. The findings should be interpreted with caution because of the cross-sectional design, modest sample size, multiple comparisons, and significant baseline differences in education and marital status between groups. Within these limits, three main observations emerged. First, clinically relevant caregiver burden was more frequent among parents of children with ASD than among controls, although the difference was not statistically significant. Second, depressive and anxiety symptoms showed trend-level elevations in the ASD group, while stress scores were similar across groups. Third, coping patterns varied according to sociodemographic characteristics, suggesting that parental adaptation is heterogeneous and influenced by family and environmental context.

### 4.1. Caregiver Burden

A higher proportion of parents in the ASD group scored above the CBI threshold for clinically relevant burden compared with controls. Although this difference did not reach statistical significance, the direction of the finding is consistent with previous literature showing that parents of children with ASD often experience sustained caregiving demands [1,2,3,19,23,24]. These demands may include supervision, management of behavioral or communication difficulties, coordination of therapy, educational advocacy, and uncertainty about the child’s future. Qualitative and cross-cultural studies have also described fatigue, financial strain, stigma, reduced social participation, and limited access to support as important contributors to perceived caregiver burden [2,3,8,23,24]. Therefore, the lack of statistical significance in the present sample should not be interpreted as evidence that burden is absent. Rather, it may reflect limited statistical power, heterogeneity within the ASD group, or characteristics of the control group that narrowed between-group contrasts.

The non-significant result is clinically important because nearly one-third of parents in the ASD group met the burden threshold. This suggests that a meaningful subgroup may require psychological support, respite, psychoeducation, or social service referral. In clinical practice, burden screening should not rely only on total scores or group averages. Item-level patterns may reveal specific domains of difficulty, such as time dependence, emotional burden, physical exhaustion, or social restriction, which can guide individualized intervention.

Importantly, exploratory adjusted analyses suggested that caregiver burden and depressive symptoms remained associated with ASD caregiver status even after accounting for differences in educational level and marital status between groups. In contrast, stress-related differences were attenuated, indicating that sociodemographic factors may partially influence stress-related outcomes in this population.

The persistence of the association between ASD caregiver status and caregiver burden may support the hypothesis that caregiving demands related to ASD extend beyond sociodemographic vulnerability alone. The need for continuous supervision, behavioral management, therapy coordination, and long-term uncertainty may contribute to sustained emotional and practical burden in these families. At the same time, the attenuation of stress-related differences suggests that certain aspects of perceived stress may be partially shaped by contextual and socioeconomic factors rather than exclusively by ASD caregiving itself.

These findings reinforce the importance of considering both caregiving-specific and sociodemographic dimensions when evaluating parental psychological functioning in ASD. They also support the use of multidimensional caregiver assessment approaches in clinical practice and future research.

### 4.2. Emotional Distress

Parents of children with ASD reported higher depressive and anxiety symptoms than controls, but these differences remained at trend level and were associated with small effect sizes. This pattern is consistent with studies reporting elevated depression, anxiety, and psychological strain among parents of children with ASD, but it also indicates that the present data do not justify strong claims of clear between-group differences [9,10,13,19]. The results are therefore better interpreted as suggesting possible elevations in internalizing symptoms among ASD caregivers that require confirmation in larger samples.

The failure of depression and anxiety differences to reach conventional statistical significance may have several explanations. The study may have been underpowered to detect small or moderate effects. The ASD group may have been heterogeneous in terms of child severity, behavioral problems, cognitive impairment, time since diagnosis, and access to services, all of which can influence parental distress [10,13,17,18,19]. In addition, the control group was recruited from the same clinical setting and may not represent a low-stress general community sample. Finally, significant differences in education and marital status between groups may have influenced emotional outcomes and limited the extent to which differences can be attributed specifically to having a child with ASD.

Stress scores were similar between groups. This finding deserves careful interpretation. One possibility is that DASS-21 stress captures recent tension, irritability, and difficulty relaxing, which may fluctuate according to short-term circumstances and may not fully capture chronic caregiving strain. Another possibility is that parents in both groups experienced comparable current stressors, particularly given that all participants were recruited in a clinical context. Previous research suggests that caregiver adaptation in ASD is shaped not only by general stress exposure, but also by support needs, coping resources, and the chronicity of caregiving demands [14,15,16,19]. Thus, depression and anxiety may have been more sensitive than stress to the chronic emotional aspects of ASD caregiving in this sample. However, this interpretation remains speculative and should be tested in future studies.

### 4.3. Coping Strategies and Sociodemographic Context

The coping results suggest that parental responses to caregiving demands are not uniform. Marital status, residence, and number of children were associated with specific coping dimensions. These findings are exploratory but clinically relevant because they indicate that caregiver support should be adapted to family context rather than delivered as a generic intervention. This interpretation is consistent with reviews showing that parents of children with ASD use a broad range of coping strategies and that these strategies are influenced by available resources, perceived control, social support, and cultural context [15,16,17,18,19].

Marital status was associated with aggressive coping, and a trend was observed for instinctive coping. This result should not be interpreted as indicating hostility or dysfunction among married parents. A more cautious interpretation is that reactive coping may emerge in families exposed to chronic pressure, role overload, and reduced recovery time. Couple dynamics, distribution of caregiving responsibilities, financial pressure, and perceived support from the partner could all influence this association. Previous qualitative studies have described interpersonal strain, exhaustion, and changes in family roles among parents caring for children with ASD [1,2,3,23]. Because the present study did not directly measure relationship quality, dyadic coping, conflict, or division of caregiving tasks, these mechanisms cannot be confirmed.

Urban residence was associated with indirect and aggressive coping. This may appear counterintuitive, as urban families may have better access to specialized services. However, urban residence may also be linked to higher occupational demands, faster pace of life, greater social comparison, more complex service navigation, or different expectations regarding child development and parental performance. Conversely, rural families may face different barriers, such as reduced access to services, transportation difficulties, or lower availability of specialized professionals. The present findings do not allow a definitive explanation, especially because confidence intervals were wide for some estimates. However, the broader literature supports the idea that coping is context-dependent and shaped by service access, stigma, social resources, and cultural expectations [2,3,8,15,16,17,20,21]. Future studies should examine not only residence as a binary variable, but also actual service access, travel burden, income, social support, and perceived stigma.

The between-group difference in seeking social support reached statistical significance, with parents of children with ASD reporting higher scores on this coping dimension than controls. This finding may suggest a greater perceived need for interpersonal or practical support in the context of ASD caregiving demands. At the same time, this result should be interpreted cautiously, as social support seeking may reflect both adaptive help-seeking and increased burden-related reliance on external resources [2,8,15,16].

### 4.4. Strengths and Limitations

A strength of this study is the simultaneous assessment of caregiver burden, depression, anxiety, stress, and coping strategies in a Romanian sample, using a comparative design. This is relevant because previous studies have often focused on either distress or coping, while fewer have integrated burden, emotional symptoms, and coping profiles within the same analysis [9,10,14,18,19]. The study also highlights sociodemographic factors that may shape coping patterns and may be clinically relevant when planning caregiver support.

Several limitations must be acknowledged. The cross-sectional design prevents conclusions about causality or directionality. The sample was relatively small and recruited from a single clinical setting, limiting generalizability. Fathers were underrepresented, which restricts interpretation of paternal coping and distress. Although exploratory adjusted analyses were conducted for educational level and marital status, residual confounding cannot be excluded, and these findings should be interpreted cautiously. No formal a priori sample size calculation was available, so the study may have been underpowered. Multiple comparisons were performed without correction, increasing the risk of type I error. Finally, the study did not include detailed clinical characterization of the children with ASD, such as severity of symptoms, cognitive impairment, behavioral problems, comorbidities, or functional level, which limits the interpretation of caregiver outcomes.

The absence of robust significant findings may be explained by several methodological and clinical factors. The sample size was modest, reducing statistical power and increasing the likelihood of type II error. The ASD group was probably heterogeneous, but the available data did not include detailed indicators such as ASD severity, intellectual functioning, language level, behavioral problems, sleep difficulties, comorbidities, therapy intensity, or time since diagnosis. These variables can strongly influence caregiver burden and emotional distress [10,13,18,19]. The control group may also have included parents experiencing health-related or family stressors, given recruitment from a clinical setting. In addition, education and marital status differed significantly between groups, and these variables may confound the relationship between ASD caregiving and parental outcomes.

The control group was recruited through voluntary online response sampling, which may introduce selection bias and limits the representativeness of the comparison group.

The relatively small sample size may have limited the statistical power to detect small or moderate between-group differences, particularly for exploratory subgroup and coping analyses.

The exploratory analysis also involved multiple comparisons. This increases the risk that some statistically significant associations occurred by chance. Therefore, significant results should be viewed as preliminary signals requiring replication, while trend-level results should be described as hypotheses rather than evidence of confirmed group differences.

### 4.5. Clinical and Research Implications

Despite these limitations, the findings suggest that caregiver assessment should be integrated into routine ASD services. Screening should include burden, depression, anxiety, and coping, rather than focusing only on general stress. Parents with elevated burden or internalizing symptoms may benefit from psychoeducation, counseling, support groups, referral to mental health services, and structured assistance in navigating educational and therapeutic resources. These recommendations are consistent with previous work emphasizing the role of support needs, social support, and coping strategies in parental adjustment to ASD caregiving [13,14,15,16].

Interventions should also consider family context. Parents with limited support-seeking, multiple children, or reactive coping patterns may require more proactive and accessible forms of support. Couple-focused interventions, emotion-regulation strategies, and family-centered planning may be useful when aggressive or instinctive coping is prominent. At the service level, improving access to reliable information, reducing stigma, and strengthening referral pathways may reduce caregiver overload [2,3,8,15,16].

Internal consistency coefficients could not be recalculated for the present sample because only aggregated subscale scores were available during secondary statistical processing, as the original dataset provided by the collaborating psychologist did not include individual questionnaire item responses. Nevertheless, the CBI, DASS-21, and SACS are previously validated psychometric instruments with established reliability in prior literature.

Future research should include larger and more diverse samples, recruit more fathers, and collect detailed information about child functioning, behavioral problems, cognitive impairment, comorbidities, therapy access, and time since diagnosis. Multivariable analyses are needed to adjust for education, marital status, residence, and other potential confounders. Longitudinal designs would be particularly valuable for clarifying whether coping strategies predict later distress or whether emotional symptoms shape coping over time [18,19].

## 5. Conclusions

This exploratory cross-sectional comparative study examined caregiver burden, emotional distress, and coping strategies among Romanian parents of children/adolescents with ASD and parents of typically developing children. Parents of children with ASD showed a higher proportion of clinically relevant caregiver burden and trend-level elevations in depressive and anxiety symptoms, while stress scores were similar between groups. However, most between-group emotional outcomes did not reach statistical significance, and observed effects were small. Therefore, the findings should be interpreted as preliminary rather than conclusive. Coping strategies appeared to vary according to marital status, residence, and number of children, suggesting that parental adaptation is shaped by family and environmental context. These associations may help identify families who need more tailored support, but they require confirmation in larger studies. The small statistically significant difference in seeking social support should also be interpreted cautiously, as its clinical magnitude was limited. Overall, the study supports the value of routine caregiver screening in ASD-related services, including assessment of burden, depression, anxiety, and coping strategies. The results also underline the need for individualized, family-centered interventions that consider sociodemographic context and available support resources. Larger longitudinal studies with multivariable adjustment and detailed characterization of child clinical features are needed to clarify the determinants of caregiver distress and to identify modifiable targets for psychosocial intervention.

## Figures and Tables

**Table 1:** Sociodemographic characteristics of parents included in the study.

Variable	ASD Group (*n* = 46)	Control Group (*n* = 46)	*p*-Value
Female sex, *n* (%)	41 (89.1%)	41 (89.1%)	1.000
Age ≥ 35 years, *n* (%)	35 (76.1%)	29 (63.0%)	0.257
Married, *n* (%)	32 (69.6%)	42 (91.3%)	0.016
Urban residence, *n* (%)	25 (54.3%)	33 (71.7%)	0.130
Higher education, *n* (%)	12 (26.1%)	35 (76.1%)	<0.001
CBI ≥ 36, *n* (%)	14 (30.4%)	8 (17.4%)	0.197

Abbreviations: ASD = Autism Spectrum Disorder, CBI = Caregiver Burden Inventory. Data are presented as mean ± standard deviation or *n* (%), as appropriate. Continuous variables were compared using Student’s *t*-test or Mann–Whitney U test, while categorical variables were compared using chi-square or Fisher’s exact tests, as appropriate.

**Table 2:** Comparative analysis of DASS-derived symptom severity scores between groups.

DASS Subscale	ASD (*n* = 44) Mean ± SD	Control (*n* = 46) Mean ± SD	Median (ASD/Control)	*p*-Value ^1^
Depression	1.80 ± 1.39	1.24 ± 0.79	1/1	0.073
Anxiety	1.75 ± 1.14	1.26 ± 0.88	1/1	0.054
Stress	1.57 ± 1.17	1.48 ± 0.94	1/1	0.861

^1^ Mann–Whitney U test (two-tailed), used due to non-normal data distribution.

**Table 3:** Comparison of Seeking Social Support (SACS) scores between study groups.

Group	Mean ± SD	Median	Range	*n*	Test	Statistic	*p*-Value
Parents of children with ASD	49.26 ± 10.47	49	28–71	46	Mann–Whitney U	U = 733.5/U′ = 1382.5	0.0114
Parents of children without health problems	44.02 ± 8.94	42	23–63	46			

Note: Data are expressed as mean ± standard deviation (SD) and median values. Non-normal distribution confirmed by KS test; comparison performed using Mann–Whitney U test; SD = standard deviation.

**Table 4:** Exploratory multivariable linear regression analyses adjusted for educational level and marital status.

Outcome Variable	Predictor	B	SE	β	*p*-Value	Model R^2^	Adjusted R^2^	F	Model *p*-Value	*n*
Caregiver burden	ASD group	12.88	4.11	0.37	0.002	0.14	0.11	4.74	0.004	92
	Educational level	8.09	3.96	0.23	0.044					
	Marital status	−4.94	4.50	−0.11	0.275					
Depression	ASD group	0.85	0.28	0.37	0.003	0.10	0.07	3.07	0.032	90
	Educational level	0.49	0.27	0.21	0.073					
	Marital status	0.23	0.31	0.08	0.458					
Anxiety	ASD group	0.66	0.26	0.32	0.011	0.08	0.04	2.38	0.075	90
	Educational level	0.35	0.25	0.17	0.166					
	Marital status	0.02	0.29	0.01	0.939					
Stress	ASD group	0.40	0.26	0.19	0.124	0.05	0.02	1.54	0.209	90
	Educational level	0.50	0.25	0.24	0.047					
	Marital status	−0.01	0.29	0.00	0.978					

Abbreviations: ASD = autism spectrum disorder; B = unstandardized regression coefficient; SE = standard error; β = standardized beta coefficient. Models were exploratory and adjusted for educational level and marital status. Predictor coding: ASD group (1 = ASD caregiver, 0 = control); educational level (1 = higher education, 0 = lower education); marital status (1 = married, 0 = unmarried/divorced).

## Data Availability

The data that support the findings of this study are available from the corresponding author upon reasonable request, subject to ethical and privacy restrictions.
